# Analysis of Stiffness of Clamped Joints versus Bolted Joints in Steel Structures by Means of Accelerometers and Shaking Table Tests

**DOI:** 10.3390/s21144778

**Published:** 2021-07-13

**Authors:** Manuel Cabaleiro, Carlos Moutinho, Cristina González-Gaya, Elsa Caetano, Victor Fco. Rosales-Prieto

**Affiliations:** 1 Department of Construction and Manufacturing Engineering, Universidad Nacional de Educación a Distancia (UNED), 28040 Madrid, Spain; cggaya@ind.uned.es (C.G.-G.); victor.rosales@ind.uned.es (V.F.R.-P.); 2 Department of Materials Engineering, Applied Mechanics and Construction, School of Industrial Engineering, University of Vigo, 36208 Vigo, Spain; 3 Construct/ViBest, Faculty of Engineering of University of Porto (FEUP), 4200-465 Porto, Portugal; moutinho@fe.up.pt (C.M.); ecaetano@fe.up.pt (E.C.)

**Keywords:** dynamic analysis, damage detection, structural SHM, clamped and bolted joints, shaking table tests, MEM accelerometers

## Abstract

This work analyzes the difference in stiffness in a steel laboratory structure using clamped joints or bolted joints and analyzes if the stiffness varies in the same way when the frame is subjected to external dynamic loads that bring the joint materials to their yield strength. To make this comparison, the differences between clamp joint and bolted joint were evaluated using a novel methodology based on the analysis of the structure’s natural frequencies from accelerometers. To perform this comparison, several laboratory tests were carried out on a frame made by clamped joints and the same frame made by bolted joints, using a set of tests on a medium-scale shake table for this purpose. The results achieved have verified the methodology used as adequate.

## 1. Introduction

Most of the structures used in industrial plants to support the facilities and machinery are made of steel. Nowadays, welding or the classic bolted joint connections are the type of joint commonly used [1,2]. Changes in the structures that support the facilities and machinery are frequent, motivated by the changes that are usual in the facilities and machinery lay-out of industrial plants. When these structures are made with welded joints, their reconfiguration is very difficult. When the structures are made with classic bolted joints, their reconfiguration is easier, since by removing the bolts, it is possible to disassemble the structure. However, in the structures made with classic bolted connections, several operations must always be carried out on the beams beforehand, such as drilling, welding of end plates, stiffening of the flanges and webs, etc. These operations made on the beams mean that each beam used in the structure is really valid only for the original structure for which it was designed and not for a new structure with a different configuration, therefore reconfiguration of classical bolt structures is really difficult [3]. 

The joints made by clamps are a promising solution that enables the manufacturing of structures that are completely removable and reconfigurable [3,4,5], avoiding that many structures manufactured with standard steel profiles have to be scrapped, allowing important economic savings and more respectful use with the environment. The main difference between clamped and classic joints is that in these new joint systems the only operation necessary during manufacturing is to cut the profile to size. This fact allows structures made with clamped joints to be completely reusable (reusing profiles and clamps). The construction of removable and reconfigurable structures is a growing trend in other areas such as modular aluminum systems [6,7] or scaffolding structures [8,9], where they are already being used. However, further development, research and tests are still necessary for commercial solutions to standard steel profiles, such as clamped joints.

**Figure 1:**
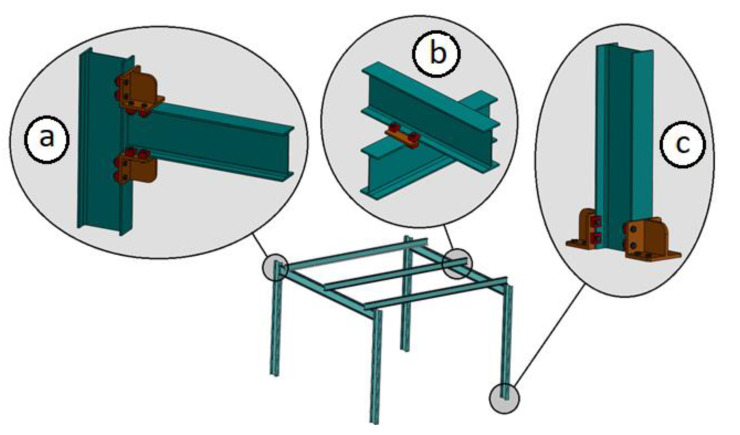
Example of different joints made with clamps: (**a**) joint of the beams end at 90° with a column; (**b**) joint of cross-joining between the flanges of the profiles; (**c**) removable base system for supports of I-type profile.

These clamps can be used to make various types of joints between standard steel profiles (Figure 1). There are currently several manufacturers of clamps for joining steel profiles [4,5].

**Figure 2:**
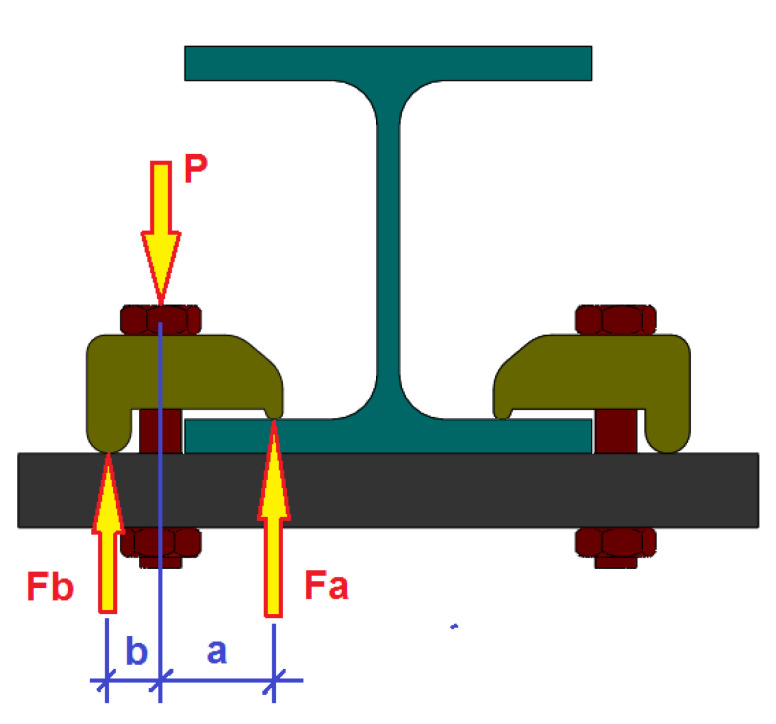
Force diagram of the operation of a clamped joint.

The clamps joint unlike the classic bolted joint works by a leaver mechanism [3]. This means that when a preload of value *P* is applied to the clamp bolt, this preload produces *F_a_* and *F_b_* reactions at the ends of the clamps (Figure 2). The reaction Fa is the force that attaches the pieces in the joint. The value of *F_a_* is proportional to the value of the force P in the bolt and *F_a_* is related to the values *a* and *b* of the clamp levers according to Equation (1):

(1) $$F_{a}=P\frac{b}{a+b}$$

where *a* is the front lever of the clamp and *b* is the rear lever. Therefore, in these types of joints with clamps for a preload *P*, a greater tightening force *F_a_* can be achieved in the joint, by varying the values of the levers *a* and *b* of the clamp.

The proper functioning of the joint directly depends on the load to which the bolts are subjected. A loss of the bolts’ preload means a loss in the clamps’ clamping force, and, therefore, it may imply a loss in the joint stiffness. Industrial structures are usually subjected to dynamic loads because they normally support machines and equipment that are in motion or have moving components. For this reason, it is important to analyze the stiffness and behavior of clamped joints with both static and dynamic loads.

The joints can be considered according to their stiffness, as rigid, semi-rigid or nominally pinned, as indicated by Eurocode 3 [10,11]. The joint may have a different degree of stiffness depending on how it was made. The different factors that affect the degree of stiffness of a bolted joint include the connected members’ stiffness, the number and arrangement of bolts as well as the bolts’ preload. 

**Figure 3:**
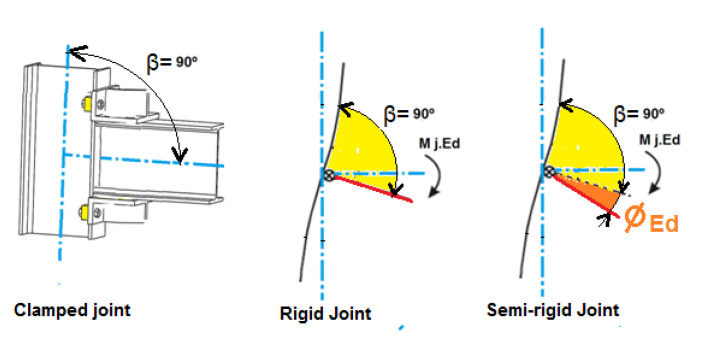
Example of rotation of a joint with initial relative angle (*β*) of 90° between the profiles for rigid and semi-rigid joint.

When a joint is not fully rigid, and a bending moment is applied to the joint, the relative angle between the connected members (*β*) that form the joint (Figure 3) varies (*φEd*). This variation affects the joint’s moment rotation diagram, thereby causing a variation of the joint’s bending moment absorption capacity and a variation of the structure’s stiffness compared to when the joints are fully rigid.

The stiffness and behavior of the classic bolted joints have already been widely studied in a large number of research works [12,13,14,15,16,17], various studies have also been carried out on dynamic stress assessment of bolted joints [18,19,20,21], but studies about clamped joints are still pending. In addition, this type of joint and its calculation is already included in various standards, such as the Eurocode 3 [10,11]. However, regarding the stiffness of clamped joints or the comparison of the stiffness of the clamped joints versus classic bolted joints, not much research work exists. Specifically with regard to clamped joints, a little bit of research has been conducted. These works include research by Cabaleiro et al. [3], which, using the T-stub methodology (according to the Eurocode 3), an axial loading study of these types of clamped joints was carried out according to the dimensions of the clamps’ lever. In that paper, a detailed study of the axial loading behavior of the clamps and of the profile is carried out. However, the analysis of their deformations or of how the bolt preload affects the joint behavior is not performed. In a subsequent work [22], the deformation behavior of these types of joints, when they are subjected to axial loading and also considering the bolt preload, has already been studied. Additionally, in the work carried out by Cabaleiro et al. [23], an analysis of the distribution of stresses in the beams’ flange was performed when a clamped joint was subjected to a bending moment. The results allowed estimation of the minimum flange length necessary in the profiles so that the clamped joint could transmit the bending moment without exceeding the profile material’s yield strength. However, none of the research work carried out to date has analyzed the behavior of this type of clamped joint with respect to dynamic stress.

The aim of the work presented here is to compare the stiffness of a frame with clamped joints versus a frame with bolted joints and analyze whether its stiffness varies in the same way when the frame is subjected to external dynamic load. To carry out this comparison and analysis, a novel methodology based on the analysis of the frame’s natural frequencies from accelerometers is used, according to the different oscillation amplitudes that are applied. For this purpose, the measurements are performed by accelerometers connected to a data acquisition system.

The use of natural frequency measurement from accelerometers as a means of measuring the variation in stiffness of steel structures has already been used in numerous works, such as the analysis on steel structure that had changes in its structural stiffness due to renovation processes [24,25]. It has also been used for the calibration of numerical models of actual steel structures and subsequent analysis of their behaviors [26,27], to the monitoring of the static and dynamic displacements of railway bridges with the use of inertial sensors [28], to measure the earthquake behavior of steel structures in the laboratory [29,30] and has also been used to dynamic assessment of masonry towers based on terrestrial radar interferometer and accelerometers [31]. The use of accelerometers for the analysis of general stiffness has also been used in various works [32,33], especially with the aim of structural health monitoring [34,35]. However, none of these works have been geared to comparing the behavior and variation of stiffness according to dynamic load by an accelerometer in a structure made with two different types of joints.

For real-time analysis and online damage identification techniques, Kalman filter solutions [36,37,38] and eigen perturbation [39,40] could be implemented. In particular, industries have introduced eigen perturbation and Kalman filter approaches along with spectral decomposition methods for condition monitoring. These solutions allow continuous remote monitoring, especially suitable for critical structures (both welded and bolted) where the response time is a critical factor, and a collapse could cause great economic or social losses. Despite the great advantages of these methodologies, in this paper a different approach was followed, which is based on simple procedure to evaluate the variation of the structural stiffness induced by damage.

The main novelties of this work are that (as far as the authors are aware) for the first time the dynamic stress behavior and the stiffness of clamped joints is analyzed, as well as, for the first time the comparison of the clamped joints with the equivalent classic bolted joints in steel structures is researched. On the other hand, a simple and effective methodology is used in laboratory tests based on the analysis of the natural frequencies of the frame using accelerometers, according to the different oscillation amplitudes that are applied using a shaking table.

In this paper, after the introduction, Section 2 describes the proposed methodology for the analysis of stiffness of clamped joints versus bolted joints. Section 3 describes the used laboratory frame, its previous numerical simulations, and laboratory tests. In Section 4 the results of the numerical simulations and the results of the subsequent laboratory tests of the frame are described. Finally, in Section 5, the conclusions are summarized.

## 2. Methods

To carry out the aim proposed in this work, the response to dynamic sinusoidal load of a frame made with clamp joints and the same frame made with bolted joints is tested by accelerometers. The frame joints are designed for both possibilities; that is, the same joint can be bolted or clamped. The frame was designed so that only the type of joint changes and the rest of the structure remains exactly the same. The tests are carried out by means of a shake table, by applying sinusoidal base loads only in the direction of the axis where the tested joints work.

In order to compare whether the stiffness of the clamped frame remains equal, higher or lower than that of the bolted frame, as well as the analysis of the behavior of its stiffness against dynamic load, the following checking methodology is used, based on natural frequency. In low damping structures, the natural frequency is a fixed value and depends only on mass and structural stiffness. When a structure is subjected to a forced movement and then released in free motion, it oscillates according to its own natural frequency of vibration. A structure can have different vibration modes and, therefore, different natural frequency values.

Each of the natural frequency modes ($\omega_{n}$) of a body is equal to Equation (2)

(2) $$\omega_{n}=\sqrt{\frac{K_{n}}{m}}$$

where *m* is the modal mass and *k_n_* is the modal stiffness.

**Figure 4:**
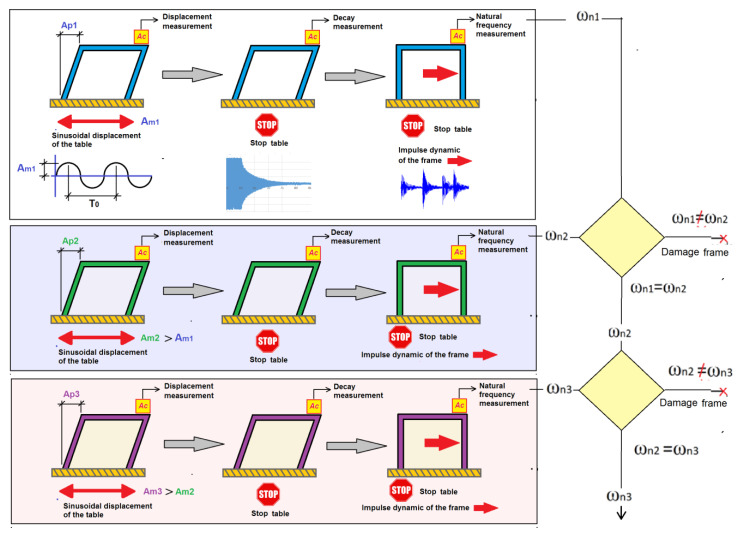
Diagram of the methodology used.

Equation (2) indicates that a structure’s natural frequency is related only to its mass and stiffness. In the tests performed in this work, the frame’s mass always remains constant; therefore, if the frame is subjected to a dynamic load that produces damage to the structure, then its stiffness will vary and, therefore, so will its natural frequency. Taking this fact into account, the frame was subjected to dynamic sinusoidal load with increasing maximum amplitude in each test, following the steps below (see Figure 4):

1. The frame is subjected to a sinusoidal ground movement of amplitude Am_1_, which will be amplified until the structure reaches an amplitude *Ap*_1_ in the frame top. *Ap*1 is the displacement value with which the frame starts being tested. The natural frequency of the frame is assumed to be the shaking table’s oscillation frequency. This way, with a small amplitude on the shaking table, a large amplitude can be obtained at the top of the structure. The frame displacements are evaluated by accelerometers located at the top of the structure, taking into account Equation (3).

(3) $$u=\frac{a}{{\left(2\pi f\right)}^{2}}$$

where *u* is the amplitude of the displacement, *a* is the maximum acceleration value measured by the accelerometer and *f* is the frequency at which the shaking table oscillates.

2. After subjecting the frame during a time (*te*) to the forced sinusoidal movement, the shaking table is stopped and the oscillation decays. The oscillation decay is measured by one accelerometer until the frame is completely stopped. During the movement’s decay, a detailed analysis of the frame behavior (especially the damping and frequency variation) is performed depending on whether the frame is bolted or clamped. To calculate the value of the damping factor during the oscillation decay, the Equation (4) is used:

(4) $$2\pi n\xi =\ln\frac{\mu_{1}}{\mu_{1+n}}$$

where *ξ* is the value of the damping factor, *n* is the number of cycles considered in its calculation, *μ*_1_ is the value of the structural response’s amplitude for period 1 and *μ*_1+*n*_ is the value of the amplitude for period 1 + *n*.

3. Once the frame is completely stopped, the frame is subjected to small manual impulses for a certain time (*t*_2_). Afterwards, the frame’s natural frequencies *ω*1*i* will be calculated with the values collected by accelerometers. The Fourier transform Equation (5) is used by means of the Pwelch function of MATLAB for calculating the natural frequency when the structure is static. The Pwelch function discretely performs the transformation by sections.

(5) $$g\left(\xi \right)=\frac{1}{\sqrt{2\pi }}\int_{-\infty }^{+\infty }f\left(x\right)e^{-i\xi x}\;dx$$

Once these steps have been carried out for a certain amplitude *Ap*_1_, the previous sequence is repeated with a higher amplitude of the sinusoidal movement *Ap*_2_. Once the frame movement with *Ap*_2_ amplitude is stopped, the natural frequency of the frame *ω*2*i* is evaluated. If the natural frequency remains the same, it means that the frame was not damaged, and its stiffness remains the same. The oscillation amplitude will continue to successively increase to a value of amplitude *Ap_n_* until the natural frequency varies. This variation in the natural frequency means that the frame is losing stiffness. The loss of stiffness in the frame indicates that it is damaged.

As already mentioned in the introduction, for real-time analysis and online damage identification techniques, Kalman filter solutions and eigen perturbation could be implemented. However, in the case of the current research where a specific evaluation is studied, such as the analysis of stiffness of clamped joints versus bolted joints in the laboratory, the methodology used in this paper (based on the analysis of the discrete data collected in several tests) is suitable.

To check for damage to the bolts of the joint and not in another part of the frame, the bolts’ tightening is checked to see if it remains unchanged after the test. When the bolt preload shows variation, it means that the bolt has been damaged. This methodology proposed in this work to detect damage to the structure is presented as suitable for cases in which the damage can happen at structure points that are difficult to measure with other devices such as strain gauges.

In a previous stage, to have reference values for the tests on the shake table, the frame was numerically modeled in order to find out three types of data:

(a) Values of natural frequencies and vibration modes.
(b) Frame displacement values that cause damage to the joint bolts.
(c) Frame displacement values that cause damage to the beams and stiffened of the flanges and webs.

## 3. Materials

**Figure 5:**
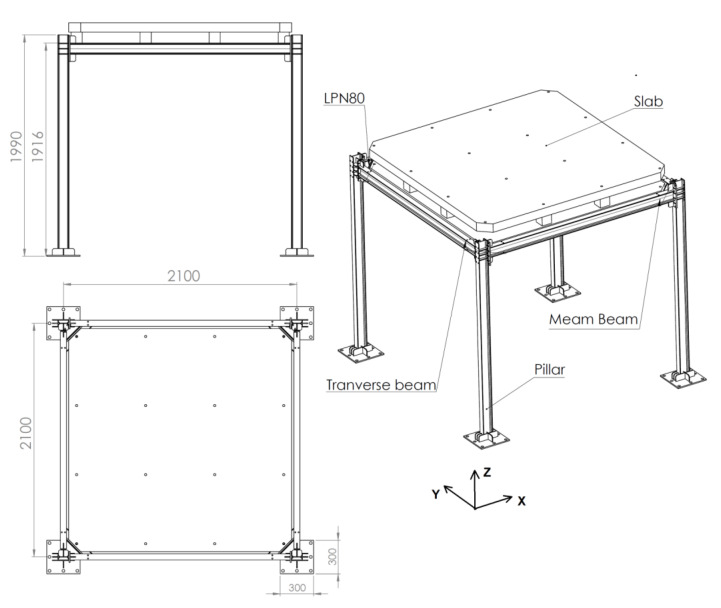
Frame used for simulations and testing.

The frame used for simulations and testing (Figure 5) is made of IPE100 steel beams, with a height of 1990 mm and 2100 mm long × 2100 mm wide. In the top part there are two concrete slabs rigidly joined together with a total mass of 1420 kg. For the connection between the profiles in the Y direction, 8 bolts M8 grade 8.8 are used in each of the joints. In the case of the other axis (X) two types of joints are used alternately: (a) the classic bolted joint, in which M8 bolts grade 8.8 are used, and (b) the clamped joint in which clamps with a width of 25 mm, thickness 10 mm and 11 mm rear lever and 11 mm average front lever are used. The bolt used with the clamps is also M8 grade 8.8.

### 3.1. Numerical Simulations

The frame’s modeling and simulation was conducted with the ANSYS^®^ version 19 software. The material used in the simulation was linear steel with elasticity behavior, Young’s modulus of 210 GPa and a Poisson’s ratio of 0.3, and specific weight of 7850 kg/m^3^ (Yield strength of beam steel 2.25 × 10^8^ N/mm^2^, Yield strength of bolt steel 6.40 × 10^8^ N/mm^2^). For the concrete, the characteristics used were Young’s modulus of 30 GPa, Poisson’s ratio of 0.18 and specific weight of 2300 kg/m^3^. For the frame’s model, 1 mm hexahedral mesh was used for the bolts and clamps. For the beams, due to its non-simple shape, a second-order tetrahedral mesh of average size 10 mm was used. For the resistant calculation, the Ansys Static Structural tool was used and the possible non-linearity of the evolution of the contact areas was taken into account in the frame. The coefficient of friction used on all contact surfaces was 0.30. Bolt preload was introduced using the Ansys Bolt Pretension tool.

The Ansys Modal Analysis tool was used to calculate the structure’s natural frequencies and vibration modes. This tool does not allow friction contacts, so the calculation was performed considering the contacts as bonded. Therefore, the result of the simulation will give the same natural frequencies for the clamped frame as for the bolted frame, while being unable to determine the difference in the frame’s stiffness according to the type of joint. The differences according to the type of joint will be determined experimentally. To calculate the frame’s resistance, due to its symmetry only half of one of the frames was used, since the model size is thus reduced to less than a quarter and, therefore, the calculation time is significantly reduced.

### 3.2. Laboratory Tests

**Figure 6:**
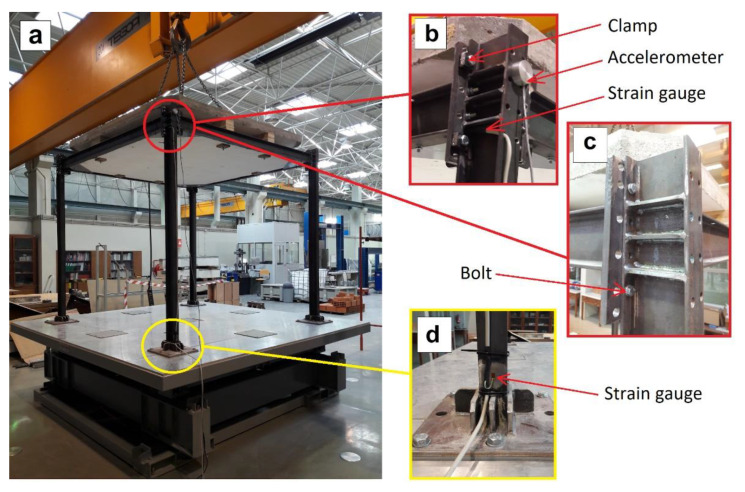
(**a**) Tested frame fastened to the shaking table; (**b**) solution of clamped joint; (**c**) solution of bolted joint; (**d**) strain gage on the bottom of frame column.

The frame used (Figure 6) is made with steel profiles of IPE100 grade S275. The shaking table belongs to ViBest laboratory (University of Porto) whose characteristics include the following: Dimensions of 3 × 3 m^2^, 2D Operation, Load capacity 10 tons, total displacement: 400 mm, maximum acceleration 0.5 g, and maximum speed 0.37 m/s. Accelerometers of type MEM model SDI 1521L were used to measure accelerations and displacements, whose characteristics include: sensitivity 2 V/g, measuring range ±2 g and noise 5 5 g√Hz. The data acquisition system is a National Instruments PXI Datalogger, which used a measurement rate of 500 Hz. The frame is rigidly fastened to the shaking table at each of the frame columns’ base plates using six M16 grade 8.8 bolts.

Strain gauges were also placed in two of the frame’s most critical points (the lower part of the frame column and in the area around the joint bolt) in order to check that the frame material’s yield strength is not exceeded, as well as to calibrate the FEM model. These critical points were determined by the preliminary numerical simulation. A torque wrench with an accuracy of 4% in the measured torque was used to control the tightening torque. Strain gauges were also placed on two bolts to check that the torque applied with the torque wrench matched the preload that was to be applied to the bolt. The strain gauges used in the frame were gauges with the following characteristics: Vishay brand, resistance 350 ± 0.3% ohm, gauge factor 2.155 ± 0.5%.

## 4. Results

### 4.1. FEM Model Results

**Figure 7:**
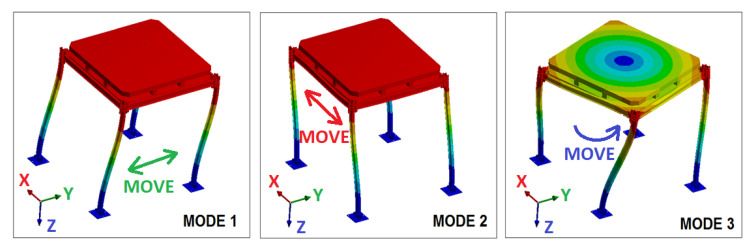
Frame’s vibration modes to be tested.

The frame’s 3D model was first subjected to a modal test with ANSYS to calculate its natural frequency values, as well as its corresponding vibration mode shapes. After calibrating the model based on a first preliminary laboratory test of the frame, three main modes of vibration were found (Figure 7): Mode 1 (displacement in the Y axis, in perpendicular direction to the joints to be tested), Mode 2 (displacement in the X axis, in the same direction as the joints to be tested) and Mode 3 (torsion with respect to the Z axis). Among these three modes, Modes 2 and 3 are the ones directly affecting the bolted or clamped joints.

While the structure has vibration modes of higher order (Table 1), they are out of the range of frequencies used to excite the frame, so they are disregarded in this study. Based on the shape of the first vibration modes, accelerometers should be placed on the top level because it is where the structure have maximum modal amplitudes. Moreover, one sensor should be located in the direction of the X axis (in the same direction as the joints to be tested) because this is the direction in which the joints work. On the other hand, the torsional vibration mode has modal components either in the X direction and Y direction, which means that it can be clearly measured with sensors positioned in both directions.

**Figure 8:**
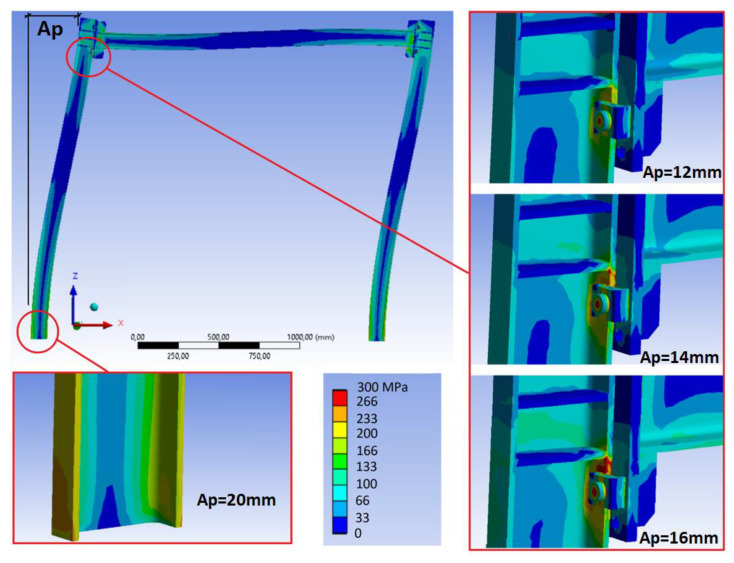
Areas of maximum stress for displacement values in the frame top of *Ap* = 12, 14, 16 mm, and the exceeded material’s yield strength at the bottom of the frame column when *Ap* = 20 mm.

Subsequently, the frame’s 3D model was subjected to a static displacement test simulation at the top of the frame with the base plate fastened, in order to find out the reference values of maximum displacements for the actual tests. Among the values analyzed are the following two:

(a) Maximum displacement value at the top of the frame from which the beams or beam plates sustain damage, that is, if they exceed the yield strength. In this test, it was found that, for the case of the bolted frame as well as for the clamped frame, the areas of maximum stress are in the area around the bolts in the frame and in the beam flange at the bottom of the frame column (see Figure 8). In the case of the clamped joint, the first stresses that exceeded the material’s yield strength in the beam stiffening area of the joints began to appear for displacements over 14 mm at the top of the frame, even though the stress was well below the yield strength in the frame columns.

When displacement values of 20 mm were reached at the top, in addition to the beam stiffening area of the joints, the material’s yield strength was also exceeded at the bottom of the frame column. In the case of the bolted frame, the situation is more favorable, not beginning to exceed the yield strength in the joints’ stiffening area until displacements reached 16 mm. Based on these results, it is determined that laboratory tests should never exceed displacement of 14 mm at the top of the frame if the idea is not to induce permanent damage to the frame members. Working with a minimum safety coefficient of 15%, the maximum values to be reached at the top of the frame would be 12 mm.

**Figure 9:**
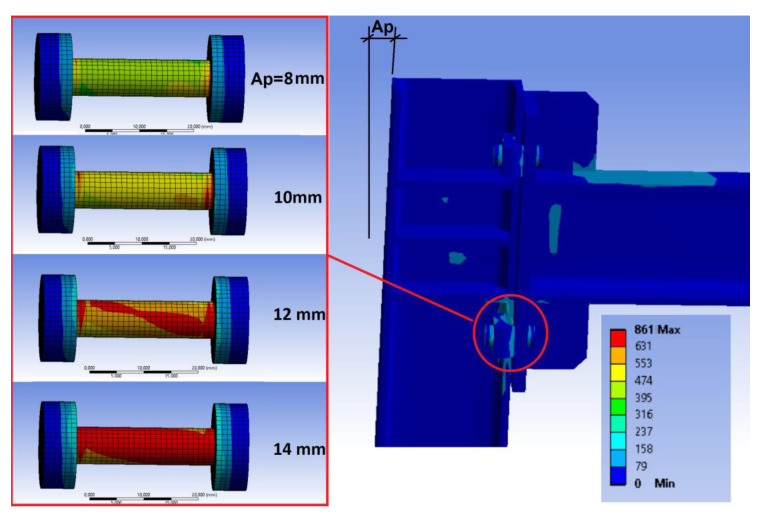
Stress distribution in the bolts of the clamped joint according to the simulation for a displacement value *Ap* at the top of the frame.

(b) Displacement value at the frame top from which the bolts or clamps sustain damage and, therefore, preload loss exists. In the results found with the simulation, it can be seen that, for the clamped frame and for a displacement value of 8 mm at the top of the frame (see Figure 9), the first results exceeding the yield strength in the bolt start to appear. With a displacement value of 10 mm at the top of the frame, some areas already appear in the bolts exceeding the material’s yield strength, but for a displacement value of 12 mm, there are already large areas of the bolt exceeding the yield strength.

It is, therefore, foreseeable that, with 10 mm displacements, the frame’s first appreciable stiffness variations appear, and that, with 12 mm displacements, the bolts are clearly damaged, and they lose part of their preload. In the case of the bolts in the frame with bolted joint, the results are similar, but the damage to the bolt appears after the 14 mm displacement at the top of the frame.

### 4.2. Results of Laboratory Tests

The frame was subjected to oscillations at the top with amplitudes close to 12 mm, but never exceeding this value. The average time to which the frame was subjected to sinusoidal movement was about 1 min, and the maximum acceleration reached at the top was about 10 m/s^2^. After stopping the frame, in order to calculate the natural frequency, the data were collected over a period of 1 min with an average of 6 manual impulses during that minute.

**Figure 10:**
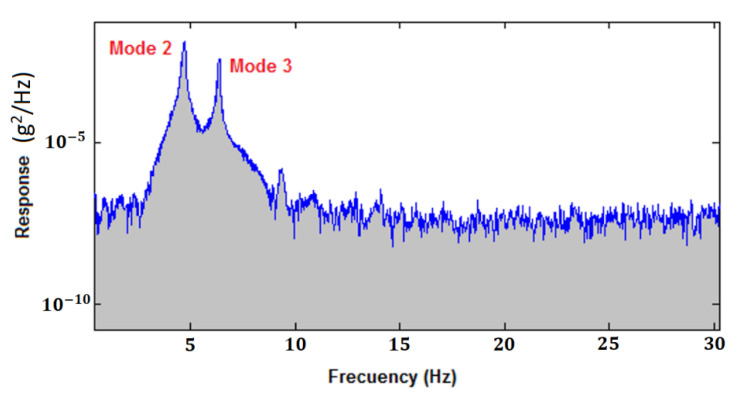
Medium spectrum of the frame response in the frequency domain for bolted joints.

In the tests carried out in the laboratory, when measuring the frame’s stiffness, it was clearly observed that the bolt frame showed higher natural frequencies (Mode 2: 4.74 Hz; Mode 3: 6.39 Hz) (Figure 10) than the clamped frame (Mode 2: 4.59 Hz; Mode 3: 6.20 Hz), which clearly indicates that the bolt frame is stiffer than the clamped frame.

**Figure 11:**
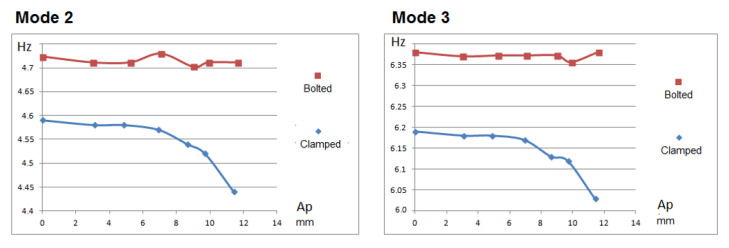
Variation of the frame’s natural frequencies after being subjected to different oscillation amplitudes at the top of the frame.

As the frame was subjected to higher oscillation amplitudes, it could be observed (Figure 11) that the frame’s natural frequencies (once finished oscillating) were kept fixed for the case of the bolted frame within a tolerance of ±0.02 Hz. However, in the case of the clamped frame, these were kept within the tolerance of ±0.02 Hz up to an oscillation amplitude of 6.5 mm. After that, a decay of the natural frequency starts being observed (but in the bolt, no preload loss occurs with the torque wrench). By contrast, between 10 and 11.5 mm of oscillation amplitude, a clear downward variation of the frame’s natural frequency is observed, which clearly indicates a loss of stiffness and, therefore, damage to the structure. In fact, this corresponds to the verification by means of a torque wrench that the bolts lost part of their preload and, therefore, the bolts were damaged.

**Figure 12:**
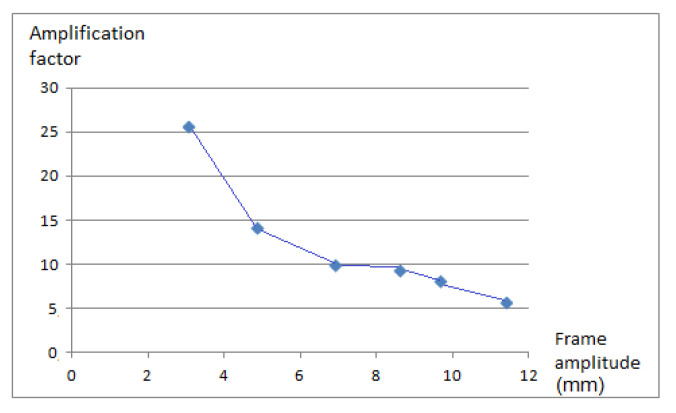
Variation of the amplification factor according to the table’s oscillation amplitude.

According to the signals collected by the accelerometer, it could be clearly seen that, as the amplitude of motion at the top of the frame varies, so, too, does the structure’s amplification factor. The amplification factor in this case is calculated as the ratio of the amplitude of the top relative to the amplitude of the motion of the shaking table. That is, with the frame beginning to swing in resonance conditions, the amplification of the motion was not proportional (Figure 12, Table 2). This indicates that the damping factor varies with the frame’s oscillation amplitude.

The stress measured with the strain gauges located on the frame column matched with those found in the FEM simulation (less than 10% difference). Regarding the strain gauges located in the area of the joint, close to the bolt where maximum stress also occurred, the data found were inconclusive. This was due to the unequal distribution of stresses that occur in that area with maximum stress peaks difficult to measure with strain gauges.

**Figure 13:**
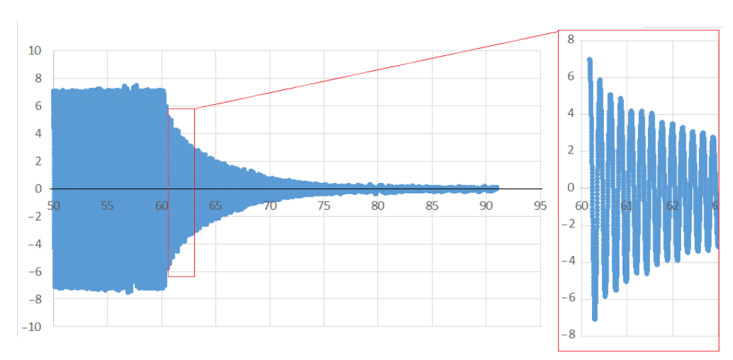
Variations in the frequency of the free oscillations that occurred after subjecting the frame to the forced sinusoidal movement and during the structure’s free oscillations.

**Figure 14:**
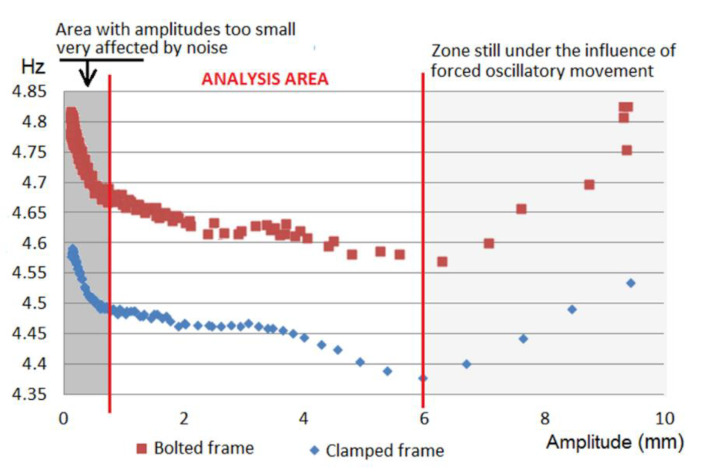
Comparison (bolted frame versus clamped frame) of the variation of the frame frequency-amplitude in free decay period after subjecting the frame to maximum oscillation.

During the structure’s free oscillations, together with the damping variation, it was also possible to clearly observe variations in the frequency of the oscillations (Figure 13) that occurred after subjecting the frame to the forced sinusoidal movement. Figure 14 shows how the frequency in the free decay period varies with the amplitude of the motion. This variation occurs very similarly in the bolted frame and the clamped frame. From these results, it follows that the variation of the frequency with the decay is related to the type of frame, slab and possibly slab/frame joint and not to the use of bolted joints or clamped joints.

**Figure 15:**
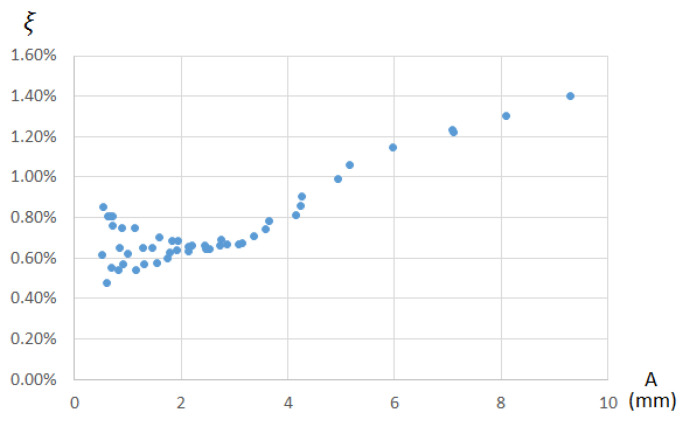
Value of the damping factor ξ according to the frame’s amplitude of free decay period in the case of clamped joints.

Figure 15 shows the value of the damping which varies with amplitude, starting at very high values when it is at high amplitudes (damping factor of 1.4% when it is at amplitudes close to 10 mm), ending in damping with average values of 0.65% for amplitudes below 3 mm.

**Figure 16:**
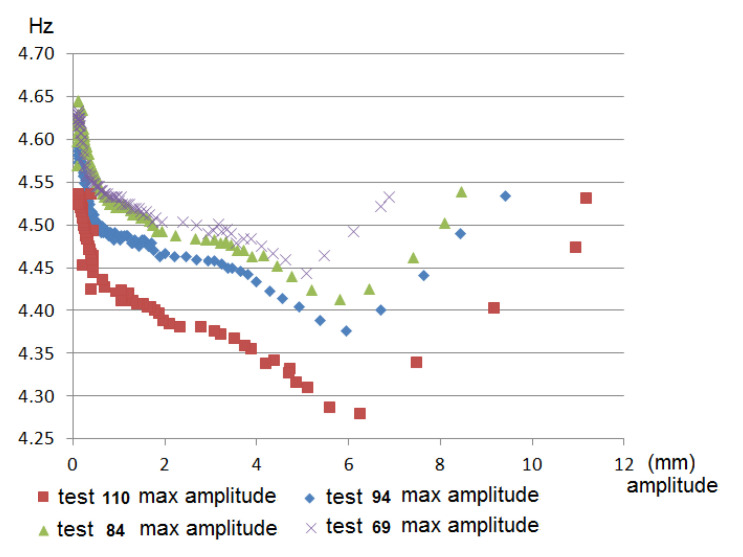
Graphs of the variation of the frame frequency–amplitude in free decay period with clamped joints for each of the tests according to the maximum amplitude at which the table was oscillated.

In Figure 16, in the case of the clamped frame, the amplitude–frequency variation graphs in the free decay period for each of the tests can be observed according to the maximum amplitude at which the frame was oscillated. As can be seen in Figure 16, the greater the frame’s maximum oscillation amplitude, the greater the damping and the lower the frequency at which the frame oscillates freely.

## 5. Conclusions

The analysis of the stiffness of clamped joints versus bolted joints by means of shaking table tests and accelerometers has been address in this work. To carry out this comparison and analysis, a methodology based on the analysis of the frame’s natural frequencies is used, according to the different oscillation amplitudes that are applied.

The proposed methodology for analyzing the behavior and damage in different solutions of structural steel joints based on the measurement of the natural frequency by accelerometers after subjecting the structure to different amplitudes of oscillatory movement has been demonstrated as adequate and simple for laboratory case studies to this. Moreover, this methodology is suitable to check for damage in structures that may be difficult to measure with other devices in the context of structural health monitoring of structures, such as those addressed in this work.

Experimentally, in the laboratory, it was clearly determined that, for the same frame with bolted joints or clamped joints, the bolted joint make the structure stiffer. Based on the results obtained with the simulation and laboratory tests, it was shown that in the case of the clamped joint with a top floor displacement amplitude of 8 mm, the joint suffered damage, while for the bolted connection a minimum displacement of 14 mm is necessary to damage the joint. Therefore, it can be concluded that to obtain the same resistance to dynamic forces, a clamped joint requires a greater bolt size or a greater number of clamps per joint, because with the same size and number of bolts the clamped joint supports lower dynamic forces (in the joint used in this work, the clamped joint supports a minimum of 42% less dynamic displacement). Moreover, based on the results obtained, it is important to recommend that for clamped structures that are going to be subjected to dynamic stresses, a prior study of their resistance to dynamic forces is always mandatory, because these forces can produce a loss of preload in the bolts and consequently a failure of the joint.

In addition, according to the results found, in a frame such as the one designed in this work that combines steel structure with a concrete slab, the damping and frequency of the assembly varies according to the frame’s oscillation amplitude. However, this variation of the damping and the frequency is not related to the type of joint used (bolted or clamped) because in both cases it varies in the same way.

The use of the numerical model allows prior estimates for better test optimization. In addition, the results obtained with the numerical simulations and with the experimental tests were very close, indicating that the numerical model used was suitable.

This work represents an important advance in the analysis of the behavior of clamped joints. Future works should study how increasing the metric of the clamped joints or the levers size of the used clamp may increase joint stiffness, making it equivalent to the corresponding bolted joint. Based on the methodology proposed here, future works could also address the detection of possible damage due to fatigue in elements of the structure when it is subjected to long periods of sinusoidal movement, based on the measurement of the natural frequency by accelerometers and shaking table tests.

## Figures and Tables

**Table 1:** Six first natural frequencies found according to FEM simulation.

Mode	Frequency (Hz)
1	1.765
2	4.780
3	6.610
4	30.471
5	51.562
6	56.804

**Table 2:** Amplification factor as the ratio of the amplitude of the frame top and the amplitude of the shaking table.

Table Amplitude (mm)	Frame Amplitude (mm)	Amplification Factor
0.12	3.07	25.6
0.34	4.84	14.2
0.70	6.91	9.9
0.92	8.62	9.4
1.20	9.68	8.1
2.00	11.40	5.7
